# Increased Crystal Field Drives Intermediate Coupling and Minimizes Decoherence in Tetravalent Praseodymium Qubits

**DOI:** 10.1021/jacs.3c02820

**Published:** 2023-08-01

**Authors:** Arun Ramanathan, Eric D. Walter, Martin Mourigal, Henry S. La Pierre

**Affiliations:** † School of Chemistry and Biochemistry, Georgia Institute of Technology, Atlanta, Georgia 30332, United States; ‡ Environmental Molecular Sciences Laboratory, Pacific Northwest National Laboratory, Richland, Washington 99352, United States; § School of Physics, Georgia Institute of Technology, Atlanta, Georgia 30332, United States; ∥ Nuclear and Radiological Engineering and Medical Physics Program, School of Mechanical Engineering, Georgia Institute of Technology, Atlanta, Georgia 30332, United States; @ Physical Sciences Division, Pacific Northwest National Laboratory, Richland, Washington 99352, United States

## Abstract

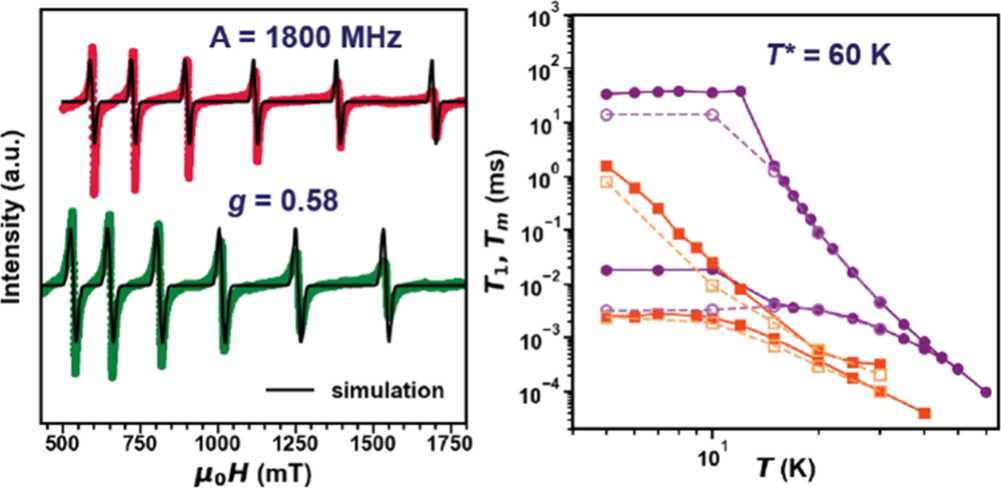

Crystal field (CF) control of rare-earth (RE) ions has been employed to minimize decoherence in qubits and to enhance the effective barrier of single-molecule magnets. The CF approach has been focused on the effects of symmetry on dynamic magnetic properties. Herein, the magnitude of the CF is increased via control of the RE oxidation state. The enhanced 4f metal–ligand covalency in Pr^4+^ gives rise to CF energy scales that compete with the spin–orbit coupling of Pr^4+^ and thereby shifts the paradigm from the ionic ζ_SOC_ ≫ *V*_CF_ limit, used to describe trivalent RE-ion, to an intermediate coupling (IC) regime. We examine Pr^4+^-doped perovskite oxide lattices (BaSnO_3_ and BaZrO_3_). These systems are defined by IC which quenches orbital angular momentum. Therefore, the single-ion spin–orbit coupled states in Pr^4+^ can be chemically tuned. We demonstrate a relatively large hyperfine interaction of *A*_iso_ = 1800 MHz for Pr^4+^, coherent manipulation of the spin with *Q*_M_ = 2Ω_R_*T*_m_, reaching up to ∼400 for **0.1Pr:BSO** at *T* = 5 K, and significant improvement of the temperature at which *T*_m_ is limited by *T*_1_ (T* = 60 K) compared to other RE ion qubits.

## Introduction

In the field of quantum information science (QIS), the fundamental unit of a quantum computer is the quantum bit or qubit, which can be placed into an arbitrary superposition of two states.^1^ Several candidates have been proposed to exhibit a two-state quantum system that can be coherently manipulated, including superconducting circuits,^2^ trapped ions,^3^ topological states in condensed matter,^4^ and electron and nuclear spins in solids.^5−7^ Interfacing between different components of a computer by using hybrid quantum systems composed of an ensemble of electron spins has been proposed as a promising route for quantum memories operating in the microwave regime.^8^ Such memories are possible by exploiting the ability to coherently manipulate the electron spins, usually implemented using various magnetic impurities, as evidenced by nitrogen-vacancy centers in diamonds, phosphorous defects in silicon,^7^ and double-vacancy sites in silicon carbide.^5,6^ Building on top of these approaches, an attractive design is to incorporate nuclear spins interacting with the electron spins via hyperfine interactions, which can offer an extra resource for storage by transfer of polarization between electron and nuclear spins and the ability to have low error rates.^9^ Furthermore, the incorporation of nuclear spins also offers the ability to scale the number of qubits by utilizing the multitude of transitions that results from the hyperfine interaction.^10^ Within this framework, rare-earth (RE) ions have been extensively studied because of their excellent coherence properties and wealth of naturally abundant nuclear spins.^11^

Paramagnetic RE ions exhibit unquenched orbital angular momentum from the atomic-like 4f states possessing electron and nuclear spins and accessible optical transitions. These properties make them attractive materials to generate a versatile quantum interface by bringing together optical and microwave addressability. As a result, a hybrid quantum system can be achieved to develop efficient and faithful microwave-optical conversion, entanglement storage, and light–matter teleportation in the telecom wavelength. In the trivalent oxidation state (RE^3+^), the core-like 4f orbitals of the RE ions are only weakly perturbed by the crystal field (CF) and minimally split the otherwise 2*J* + 1 fold-degenerate free-ion ground-state (GS) ^2*S*+1^L_*J*_. This electronic structure results in rich physics and has been used to design new quantum materials with emergent phenomena.^12−14^ Therefore, in RE ions, the CF states can act as qubit states.^11^ Among the RE ions, Pr^3+^,^15^ Nd^3+^,^16^ Er^3+^,^11^ and Yb^3+^^17,18^ have been of primary interest. Recently, synthetic chemistry has been proposed to offer tunability of quantum states by engineering the ligand field experienced by the electron spin in the form of molecular qubits.^19−21^ Besides tuning the ligand field, synthetic chemistry also offers the ability to engineer the electronic structure of the RE ion by providing control over the formal oxidation state of the metal center, as evidenced in recently explored reduced RE molecular complexes of La^2+^ and Lu^2+^. In these systems, a single unpaired electron resides in an orbital with a mixed 5d/6s character rather than the 4f orbital, giving rise to a clock transition and enhanced coherence.^22,23^

**Figure 1:**
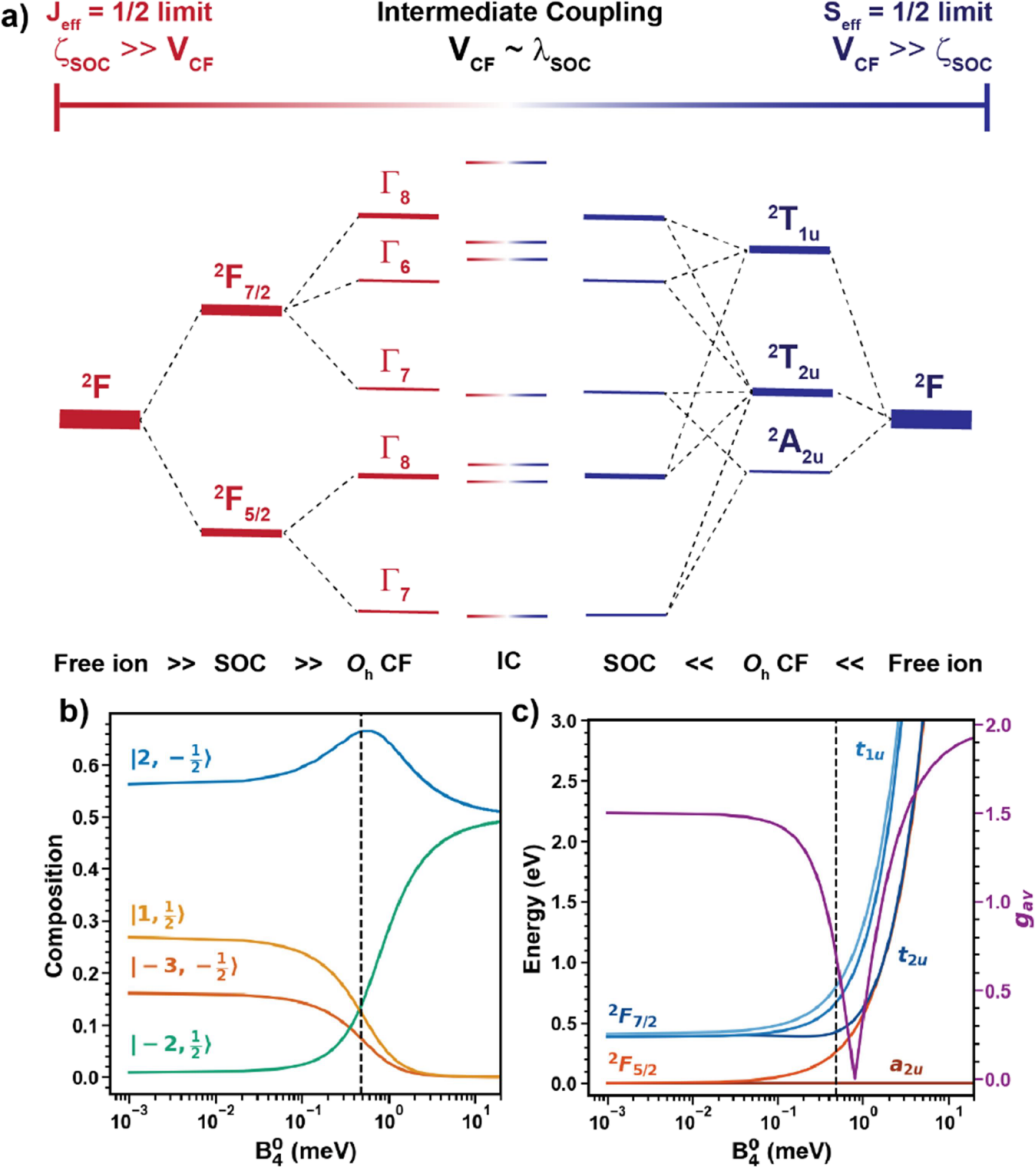
(a) Energy splitting diagram of a 4f^1^ ion from ζ_SOC_ ≫ *V*_CF_ limit (left) and ζ_SOC_ ≪ *V*_CF_ limit (right) with the IC regime in the middle. (b) Composition of the GS wavefunction in Pr^4+^ as a function of *B*_4_^0^. (c) Evolution of the eigen energies of the CF states and *g*_av_ in Pr^4+^ as the paradigm shifts from ζ_SOC_ ≫ *V*_CF_ limit to the ζ_SOC_ ≪ *V*_CF_ limit. The black line in (b) and (c) shows the position of BaPrO_3_ based on our CF calculations.

While 3+ is the most stable oxidation state for RE ions, synthetic chemistry has pushed the boundaries of RE ions by accessing the unusually high 4+ oxidation state in Ce, Pr, and Tb.^24−28^ Recently, we showed that Pr^4+^ ions exhibit an unusually large CF energy scale, almost an order of magnitude greater than its 3+ counterparts, and established that the traditional ionic paradigm, used to describe Ln^3+^ ions, breaks down for Pr^4+^ due to hybridization of the Pr-4f electrons with the ligand valence electrons (analogous to transition metals as shown in Figure 1a).^29^ Following this observation, it is enticing to use high-valent RE ions like Pr^4+^ as an alternative or compliment to Ln^3+^ systems to build novel quantum architectures with long-lived quantum memories.

A key ingredient in determining the coherence time of an electron spin is the spin–lattice relaxation time, *T*_1_, that is strongly temperature-dependent.^30^ The relaxation dynamics of *T*_1_ arises from the interplay of direct, Raman, and Orbach processes, which enable the exchange of energy between the spin system and lattice bath when the electronic energy levels (CF states for RE ions) are modulated.^31^ Suppressing the efficacy of the Raman and Orbach processes driven by acoustic and optical phonons, respectively, is key to establishing long coherence times. The intuitive approach to minimize spin–phonon coupling is engineering of lattice vibrational modes and can be achieved by judicious choice of the host lattice/ligand architecture.^32−34^ However, the chemical properties that drive *T*_1_ remain an open question and demonstrate that there is a rich chemical space still to be explored for QIS applications.

Alternative strategies for RE ions include CF control usually achieved by careful choice and control of symmetry and use of ions with an S-like electronic GS, where the vanishing orbital angular momentum (μ_orb_/μ_spin_ ≈ 0) suppresses spin–phonon coupling. Within this framework, Pr^4+^ ions are advantageous, given that the first CF excited state is at ∼2000 cm^–1^ compared to a few hundreds of cm^–1^ observed for other Ln^3+^ ions.^35^ Furthermore, the large CF energy scale competes with spin–orbit coupling (SOC), which mixes the excited-state SOC multiplet into the GS and thereby minimizes the orbital momentum (as observed from X-ray magnetic circular dichroism measurements: μ_orb_/μ_spin_ ≈ 1.8 for Pr^4+^ compared to μ_orb_/μ_spin_ ≈ 8 for Ce^3+^).^35^ Pr^4+^ also has a very small *g* value (*g*_av_ < 0.8), one of the smallest among the RE ions, which offers the ability to suppress decoherence from spectral diffusion (SD) due to magnetic dipolar interactions. As a bonus, Pr has a very large nuclear spin, ^141^Pr (100% natural abundance with *I* = 5/2), which can further be used for coherent manipulation via hyperfine interactions.

**Figure 2:**
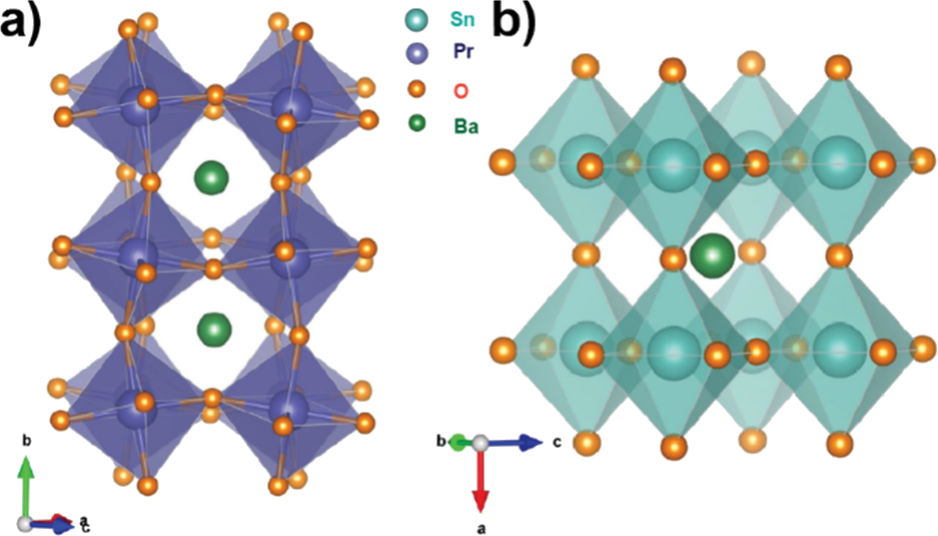
(a) Crystal structure representation of BaPrO_3_. (b) Crystal structure representation of the host lattice BaSnO_3_.

In this work, we use the anomalously large CF splitting of Pr^4+^ to avoid electronic excitations overlapping with the vibrational density of states. This electronic manifold is achieved by using chemical design principles to stabilize Pr in its unusually high 4+ oxidation state by a judicious choice of oxide host lattices.^28^ The evolution of single-ion GS wavefunction and properties of Pr^4+^ as the paradigm shifts from ζ_SOC_ ≫ *V*_CF_ to ζ_SOC_ ≪ *V*_CF_ is considered using a toy model to evaluate the unique ability to tune the spin–orbit coupled single-ion states in Pr^4+^-based systems. We demonstrate the single-ion electronic structure of Pr^4+^ in a six-coordinate perovskite lattice, BaPrO_3_, by using a combination of thermo-magnetic measurements and CF theory. Coherence studies on Pr^4+^ doped in BaZrO_3_ and BaSnO_3_ host lattices using a combination of continuous-wave (CW) and pulsed X-band EPR measurements lends credibility to our design strategy and establishes Pr^4+^ as a potential candidate for QIS applications.

## Results and Discussion

Crystalline powder samples of BaPrO_3_, Pr:BaSnO_3_ (2% doping: **2Pr:BSO**; 0.1% doping: **0.1Pr:BSO**), and Pr:BaZrO_3_ (2% doping: **2Pr:BZO**; 0.1% doping: **0.1Pr:BZO**) were synthesized using traditional solid-state reactions (see Supporting Information), and phase purity was confirmed using powder X-ray diffraction (Figure S1). The parent compound, BaPrO_3_, crystallizes in an orthorhombic *Pnma* space group^36,37^ different from the host materials (BaMO_3_; M = Sn, Zr) which crystallize in a cubic, ideal perovskite *Pm*3̅*m* structure, as shown in Figure 2.^38^ The orthorhombic distortion in BaPrO_3_ is due to cooperative buckling of the corner sharing octahedra with respect to each other, resulting in reduction of local symmetry at the B site from *m*3̅*m* (*O*_h_) in the host materials to 1̅ in the parent compound. However, the PrO_6_ octahedra in BaPrO_3_ are very close to a perfect *O*_h_ with only small changes in the nearest-neighbor oxygen coordination. Therefore, BaPrO_3_ is an ideal model compound to study the single-ion electronic structure of Pr^4+^ in the PrO_6_ moiety and to understand the microscopic origins of coherent spin dynamics of ^141^Pr^4+^ ions in BaMO_3_ (M = Zr, Sn) host lattices. It should be noted here that BaMO_3_ host lattices were chosen to provide stabilization of the 4+ oxidation state and to obtain phase pure compounds. Experiments to design host lattices with minimal nuclear spins in the surrounding bath (in materials such as La_2_M_2_O_7_ (M = Sn, Zr)) were not fruitful as phase segregation was observed.

As reported by our group and others,^35,39^ Pr^4+^ exhibits an unusually large CF splitting which competes with the SOC, yielding drastically different single-ion properties than expected in the ζ_SOC_ ≫ *V*_CF_ limit as shown in Figure 1a, and requires an intermediate coupling (IC) scheme to describe the GS properties.^35,39,40^ Pr^4+^ is a Kramers ion with a 4f^1^ electron configuration and couples the electron spin, *S* = 1/2, and orbital angular momentum, *L* = 3, to give rise to a *J* = 5/2 GS (^2^F_5/2_) and a *J* = 7/2 excited state (^2^F_7/2_) in the |*j*, *m*_*j*_⟩ basis. The GS Kramers doublet (KD) is given by , where α^2^ ∼ 1/6. In this framework, the CF Hamiltonian (*Ĥ*_CF_) is diagonalized only within the ^2^F_5/2_ SOC manifold as is the case for traditional trivalent Ce^3+^ systems.

**Figure 3:**
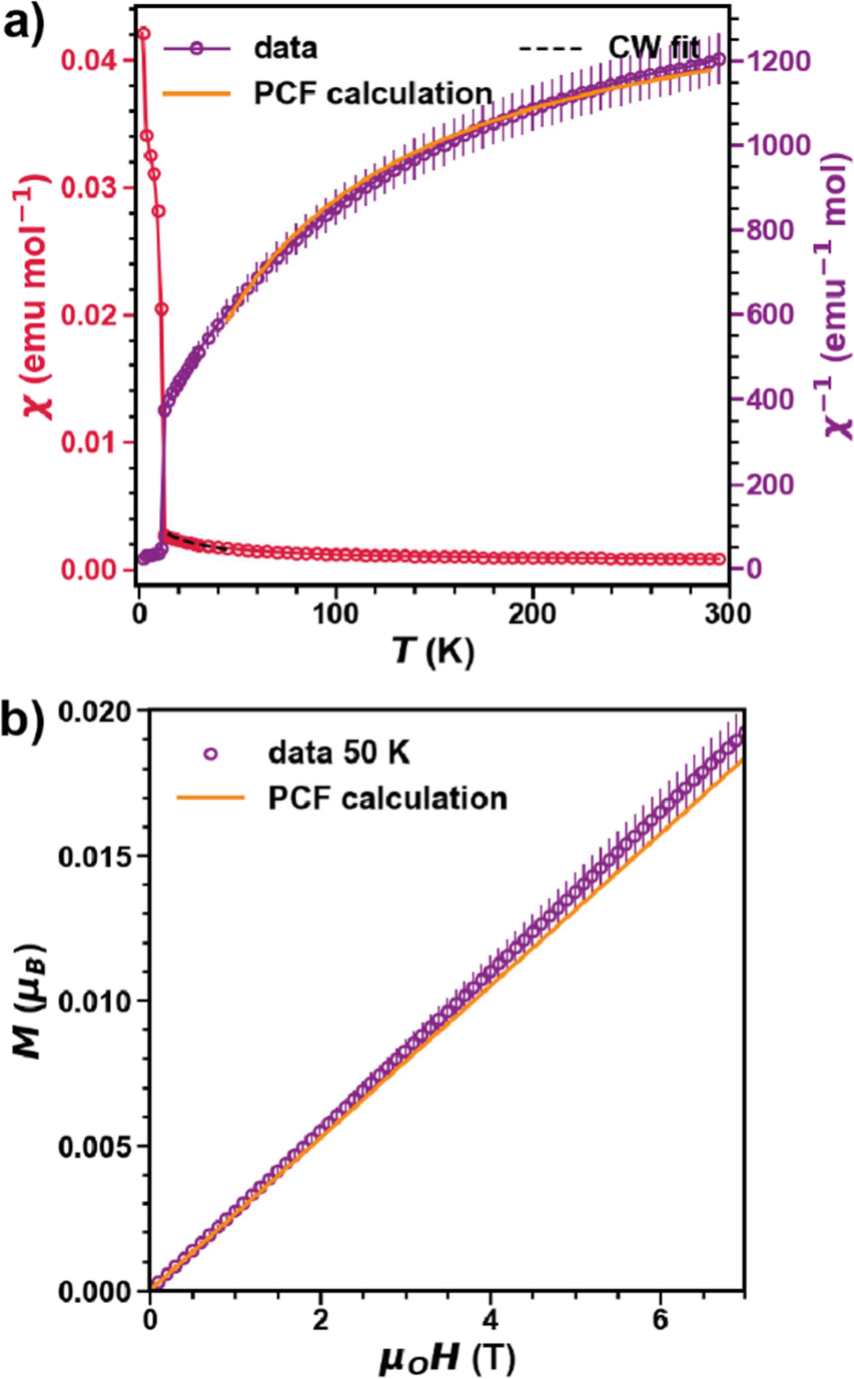
(a) Magnetic susceptibility (χ(*T*)) and inverse susceptibility (χ(*T*)^−1^) data of BaPrO_3_ measured under μ_0_*H* = 0.1 T plotted together with the CF model and a Curie–Weiss analysis in the temperature range 10 < *T* < 40 K. (b) Isothermal magnetization *M*(*H*) at *T* = 50 K for BaPrO_3_ plotted together with the CF model. *T* = 50 K was chosen so that BaPrO_3_ is well above the ordering temperature and free from short-range correlations.

Since *j* is not a good quantum number in the IC regime, the |*m*_l_, *m*_s_⟩ basis can be used to describe the electronic structure of Pr^4+^. In the |*m*_l_, *m*_s_⟩ basis, the *O*_h_ CF splits the seven 4f orbitals to GS a_2u_ and excited triply degenerate t_1u_ and t_2u_ states. In the presence of SOC, the seven 4f orbitals mix, yielding seven KD. In the |*m*_l_, *m*_s_⟩ basis, the nature of the GS KD is given as . The first two components of Γ_7_^LS^ KD (*m*_l_ = −3, −2) are derived from , , states in the |*j*, *m*_*j*_⟩ basis, while the last components (*m*_l_ = 1, 2) are derived from ,  states. Within this framework of IC, the *Ĥ*_CF_ is diagonalized using the entire set of 14 |*m*_l_, *m*_s_⟩ states.

To understand the implications of the IC scheme, the evolution of the single-ion properties of Pr^4+^ is studied in the model Hamiltonian:
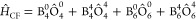
1where *B*_*n*_^*m*^ are the fourth- and sixth-order terms and *Ô*_*n*_^*m*^ are the corresponding Stevens operator equivalents,^41^ constrained by the *O*_h_ symmetry of an isolated PrO_6_ unit. The eigen energies, GS wavefunction composition, and *g*_av_ as a function of *B*_4_^0^ for fixed values of *B*_6_^0^ are calculated as shown in Figure 1b,c (see also Supporting Information). With *B*_6_^0^ = −0.004, for small values of *B*_4_^0^, the system can be considered as a traditional trivalent lanthanide where the ζ_SOC_ ≫ *V*_CF_ limit still applies. The eigen states split as expected for the *O*_h_ CF (Figure 1c). As *B*_4_^0^ increases, the paradigm shifts from ζ_SOC_ ≫ *V*_CF_ limit to ζ_SOC_ ≪ *V*_CF_. For nonphysically large values of *B*_4_^0^, the eigen states relax to three sets of 4f orbitals a_2u_, t_2u_, and t_1u_ as expected in the ζ_SOC_ ≪ *V*_CF_ limit. Looking at the composition of the wavefunction (Figure 1b), it is evident that with an increase in CF energy scale, *m*_l_ = +1, −3 states begin to decrease from the original Γ_7_ KD as the system relaxes to *m*_l_ = ±2 states corresponding to the a_2u_ GS. The shift in paradigm between the two limits significantly impacts the single-ion electronic structure, evident from the consistent change in *g*_av_ of the system. Within this framework, among the RE ions, Pr^4+^ offers the unique ability to tune the spin–orbit coupled wavefunction for a given symmetry by its ability to access the IC regime. It is important to note that the quenching of the orbital angular momentum is a product of the IC regime (Figure 1a–c). In either the CF or SOC limits, the orbital angular momentum is partially or completely recovered.

BaPrO_3_ exhibits a magnetic transition at *T*_N_ ∼ 11 K observed in χ(*T*), as shown in Figure 3a.^37^ Curie–Weiss analysis in the 10 < *T* < 40 K range yields θ_CW_ ∼ −35 K and μ_eff_^CW^ = 0.75(2) μ_B_, which is significantly lower than the expected value for a free *f*^1^ ion (2.54 μ_B_). All CF excitations for Pr^4+^ in BaPrO_3_ (measured using optical spectroscopy) have been reported previously with *E*^1^ = 0, *E*^2^ ≈ 249.5, *E*^3^ ≈ 252, *E*^4^ ≈ 389, *E*^5^ ≈ 655, *E*^6^ ≈ 657, and *E*^7^ ≈ 818 meV.^47^ As expected, Pr^4+^ exhibits an unusually large *V*_CF_ energy scale comparable to the ζ_SOC_ ≈ 112 meV,^48^ and therefore, the single-ion properties must be modeled in the IC regime as described earlier. The single-ion CF Hamiltonian for Pr^4+^ can be written in a truncated symmetry basis, as shown in eq 1. The CF Hamiltonian, *Ĥ*_CF_^Pr^, is then fit to the observed eigen energies from optical measurements, their corresponding degeneracies, and magnetic susceptibility data at μ_o_*H* = 0.1 T above 40 K (*T* > 40 K was chosen to avoid short-range correlations) as shown in Figure 3a with a fixed value of ζ_SOC_ ≈ 112 meV. This analysis yields a set of new KD’s with the GS wavefunction expressed as  with *g*_av_^CF^ = 0.68 comparable to the value extracted from the CW fits. The resulting model reproduces magnetization at *T* = 50 K, as shown in Figure 3b. This analysis clearly shows that the GS of Pr^4+^ deviates significantly from the expected *V*_CF_ ≪ ζ_SOC_.

**Figure 4:**
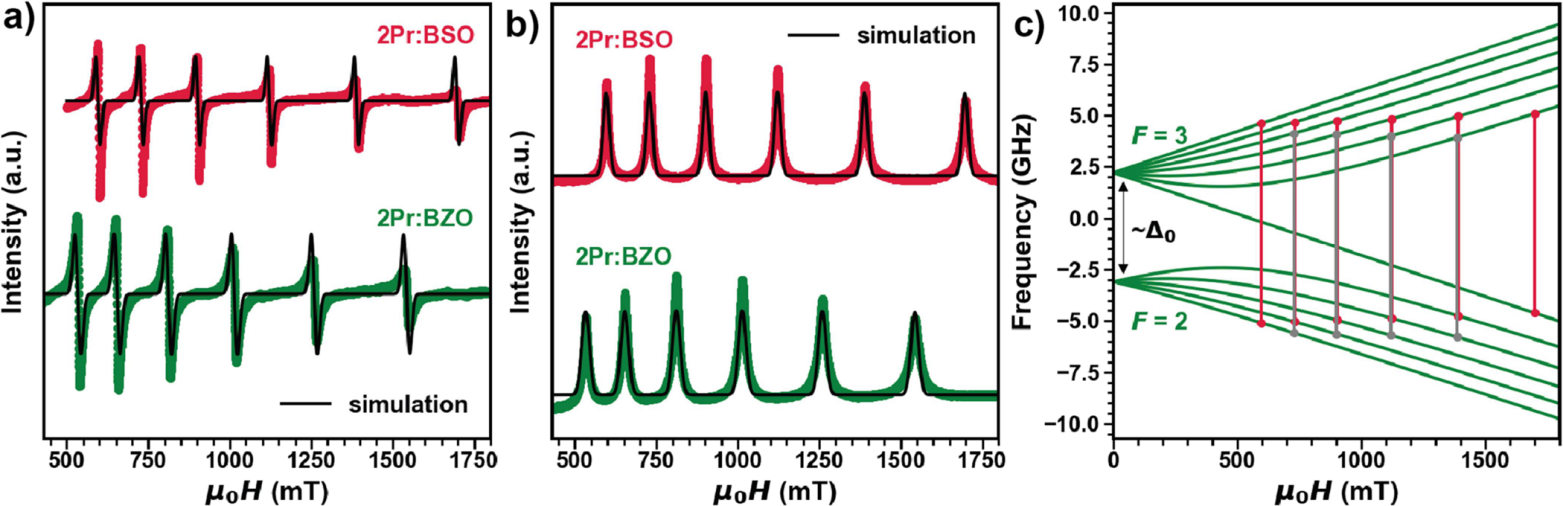
(a) CW X-band EPR of **2Pr:BSO** measured at *T* = 5 K and the corresponding simulation. (b) echo-detected field-swept (EDFS) spectra of **2Pr:BSO** and **2Pr:BZO** measured at *T* = 5 K and the corresponding simulation. (c) Simulated energy diagrams of Pr^4+^ for the *B*_0_∥*Z* orientations of the applied magnetic field calculated with parameters extracted from **2Pr:BSO**. Given that only isotropic values were used for calculations, there was no orientation dependence for the energy diagrams.

Having established the single-ion electronic structure of Pr^4+^ in a perovskite ABO_3_ lattice, the relaxation and coherent spin dynamics of electron and nuclear spins of ^141^Pr^4+^:BaMO_3_ (M = Zr, Sn) were investigated using CW and pulsed EPR at the X-band (*f* = 9.4 GHz and *B*_0_ < 1.8 T). Figure 4a shows the CW-EPR spectra for **2Pr:BSO** and **2Pr:BZO** measured at *T* = 5 K, revealing a six-line pattern due to the unpaired electron and its hyperfine coupling with the *I* = 5/2 ^141^Pr isotope. The EPR spectrum was analyzed using an effective spin Hamiltonian of eq 2 describing a lone *S* = 1/2 electron coupled to an *I* = 5/2 nuclear spin:
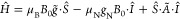
2where the first two terms denote electron and nuclear Zeeman interactions, *g̃* is the *g*-tensor, the third term represents the electron–nuclear hyperfine interaction, and *Ã* is the hyperfine coupling tensor. The X-band EPR simulations (using the MATLAB toolbox EasySpin)^49^ also shown in Figure 4a yield a *g*_iso_^EPR^ ≈ 0.57 comparable to *g*_iso_^CF^ and a large hyperfine interaction of *A*_iso_ ≈ 1771 MHz. At zero field, where the hyperfine interaction is the strongest, the nuclear spin and electron spin couple, yielding two states with total angular momentum *F* = *I* ± *S* = 5/2 ± 1/2 = 2 and 3 separated by , as shown in Figure 4c. Figure 4c also shows the six allowed EPR transitions (red lines) expected for ^141^Pr^4+^ and the corresponding forbidden transitions (gray lines) calculated with the parameters extracted for **2Pr:BSO**. **2Pr:BZO** yields very similar results with *g*_iso_^EPR^≈ 0.63 and *A*_iso_ ≈ 1789 MHz as shown in Figure 4a and are tabulated in Table S6.

**Figure 5:**
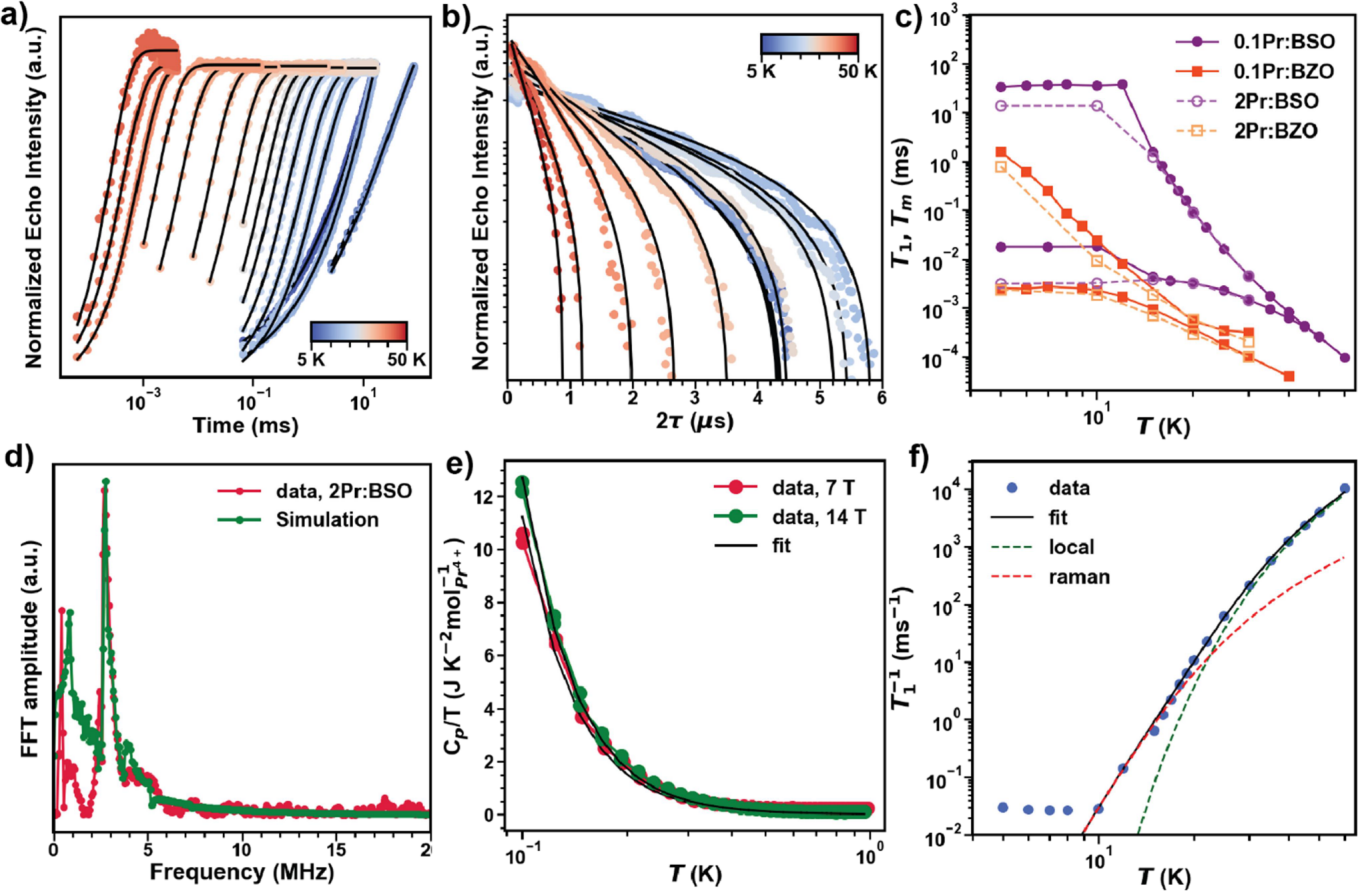
(a) Inversion recovery traces and the corresponding fits used to extract *T*_1_ for **0.1Pr:BSO** measured at different temperatures. (b) Echo decays and the corresponding fits used to extract *T*_m_ for **0.1Pr:BSO** measured at different temperatures. (c) *T*_1_ and *T*_m_ as a function of temperature for different Pr^4+^ systems studied. (d) Frequency domain data of 3P ESEEM on **2Pr:BSO** measured at *T* = 5 K and μ_0H_ = 597.2 mT and the corresponding simulation showing the Lamour frequency of ^135^Ba at the field measured. (e) Specific heat data measured for **2Pr:BSO** at *T* < 1 K and μ_0H_ = 7 and 14 T showing the Schottky anomaly corresponding to hyperfine interaction in Pr^4+^ and the corresponding fits. (f) Spin–lattice relaxation rate of **0.1Pr:BSO** as a function of temperature and the fits corresponding Raman and local mode-based decoherence mechanisms.

Probing the spin dynamics of ^141^Pr^4+^ with pulsed EPR methods, the echo-detected field-swept (EDFS) spectrum of **2Pr:BSO** was recorded by monitoring the integrated spin echo intensity as a function of applied dc field using the two-pulse echo sequence (π/2 – τ – π – τ – *echo*) with τ = 120 ns and is shown in Figure 4b. The spectrum reveals six broad transitions consistent with CW-EPR. Modeling the spectrum with the parameters extracted from CW-EPR yields good agreement with the experimental data (Figure 4b). **2Pr:BZO** yields very similar results in good agreement with CW data as shown in Figure 4b and are tabulated in Table S6. Electron spin relaxation, characterized by the spin–lattice relaxation time constant, *T*_1_, is commonly caused by spin–phonon coupling and, in most cases, limits the coherence times of the electron spin. Furthermore, *T*_1_ also affects decoherence indirectly, where the spin flips of neighboring electron spins lead to SD of the central spin.^18^ Therefore, the *T*_1_ of ^141^Pr^4+^ ions in the temperature range 5–60 K was studied using the inversion-recovery method (π – τ_r_ – π/2 – τ_e_ – π – τ_e_ – *echo*), where τ_r_ is swept, as shown in Figure 5a. These experiments, at an applied field of *B*_0_ = 592.7 mT (**2Pr:BSO**) and 530.8 mT (**2Pr:BZO**), focus on the field of the largest intensity echo. The resulting saturation recovery traces were fit with a standard stretched mono-exponential function (see Supporting Information), and the extracted *T*_1_ values are plotted in Figure 5c as a function of temperature. At low temperatures (<12 K), *T*_1_^**2 Pr:BSO**^ shows weak temperature dependence, reaching a maximum of ∼13 ms at 5 K, while *T*_1_^**2 Pr:BZO**^ shows a strong temperature dependence, reaching a maximum of ∼0.7 ms at 5 K, comparable to other oxide host lattices.^17,18^ In either case, the electron spin–lattice relaxation rate in the low-temperature regime is inversely proportional to temperature which can be attributed to a direct one-phonon process.^18^ At high temperatures (>12 K), *T*_1_ begins to precipitously decrease, reaching a value of *T*_1_^**2 Pr:BSO**^ = 4.65 μs and *T*_1_^**2 Pr:BZO**^ = 0.31 μs at *T* = 30 K. In order to understand the effects of dipolar magnetic interactions, further diluted samples with a Pr^4+^ concentration of ∼0.1% were analyzed. Dilution to ∼0.1% improves *T*_1_, reaching a maximum of *T*_1_^**0.1 Pr:BSO**^ ≈ 33 and *T*_1_^**0.1 Pr:BZO**^ ≈ 16 ms for the BSO and BZO host lattices at *T* = 5 K, respectively. 0.1% dilution improved *T*_1_ only for *T* < 12 K, as shown in Figure 5c, consistent with direct processes from spin–spin-based dipolar interactions being the dominant decoherence mechanism. For *T* > 12 K, *T*_1_ for 0.1% dilutions overlaps with 2% dilutions for both host lattices, indicating that decoherence is not limited by dipolar interactions with additional decoherence mechanisms coming in to play.

In this higher temperature regime, two-phonon processes characterized via a combination of resonant (Orbach), nonresonant (Raman), and local modes dominate.^50^ The Orbach process dominates when the temperature is sufficient to excite phonons that resonate with an excited state (in this case CF states). Given the first CF excited state is ∼2000 cm^–1^, Orbach processes should have little effect on the relaxation. Therefore, the temperature dependence of *T*_1_^**0.1 Pr:BSO**^ for *T* > 10 K was fit to a combination of Raman and local modes based on a general description of the two-phonon process which takes into account the maximum phonon energy *k*_b_θ_D_, where θ_D_ is the characteristic Debye temperature (Figure 5f and further details in Supporting Information). The data suggests a θ_D_ = 180 K consistent with the IR-phonon spectra of BaSnO_3_ which identifies the lowest optical phonon mode at 135 cm^–1^ (195 K).^51^

Having now established the bounds on spin coherence lifetimes from spin–lattice relaxation, the lifetimes of the coherent superposition state of the qubit, parameterized by phase memory time *T*_m_, were measured through two-pulse Hahn echo measurements. The echo intensity was measured as a function of 2τ as shown in Figure 5b and clearly shows an exponentially decaying signal. *T*_m_ was extracted by fitting the data to a standard mono-exponential function, yielding *T*_m_^**2 Pr:BSO**^ = 3.1 and *T*_m_^**2 Pr:BZO**^ = 2.3 μs at *T* = 5 K. *T*_m_^**2 Pr:BSO**^ follows *T*_1_^**2 Pr:BSO**^ with weak temperature dependence, while *T*_m_^**2 Pr:BZO**^ shows weaker temperature dependence compared to *T*_1_^**2 Pr:BZO**^ in the temperature range *T* < 12 K. Similar to *T*_1_, *T*_m_ improved on dilution to 0.1%, reaching a maximum of *T*_m_^**0.1 Pr:BSO**^ = 18 and *T*_m_^**0.1 Pr:BZO**^ = 2.5 μs at *T* = 5 K for both host lattices and begins to decrease at higher temperatures, reaching *T*_m_^**0.1 Pr:BSO**^ = 0.26 and *T*_m_^**0.1 Pr:BZO**^ = 0.1 μs at *T* = 60 K with minimal effects of dilution for *T* ≳ 12. *T*_m_ is bound by *T*_1_ above T* = 40 and 60 K for **0.1Pr:BZO** and **0.1Pr:BSO**, respectively. Note that at low temperatures, the echo decay curves show strong electron spin-echo envelope modulation (ESEEM) from Ba nuclear spins (vide infra). The maximum *T*_m_ value extracted for ^141^Pr^4+^ is one of the largest among the RE ions (Table 1). Furthermore, coherent spin dynamics are detectable up to T* = 60 K in **0.1Pr:BSO**, which is greater than all RE ions except Gd^3+^. This phenomenon is attributed to the high-energy first electronic state in both Pr^4+^ and Gd^3+^. However, in Gd^3+^ the excited state is a SOC multiplet, whereas in Pr^4+^, the excited state is purely CF derived (Table 1). We note here that relaxation measurements at other hyperfine transitions also yield very similar results (see Table S5 and Figures S4 S5).

**Table 1:** Comparison of Parameters Extracted for Pr^4+^ from This Work with Other 4f-Based RE Ions^a^

RE ion	host lattice	excited state (cm^–1^)	*A*_*J*_ (MHz)	*g*_av_	*T* (K)	*T*_1_ (ms)	*T*_m_ (μs)	T* (K)	ref
Pr^4+^	BaSnO_3_	∼2000	∼1800	∼0.58	5	∼33	∼18	∼60	this work
Ce^3+^	CaWO_4_	53–135	N/A	∼1.4	5	∼24.7	∼14.2	∼18	(42)
Nd^3+^	Y_2_SiO_5_	∼77	∼530	∼2.6	5	∼30	∼106	∼7	(16)
Gd^3+^	CaWO_4_	∼33,000^d^	∼15	∼2	6	∼8	∼5.4	∼70	(43)
Gd^3+^	trensal^b^	∼33,000^d^		∼2	5	∼0.03	∼12	>30	(44)
Gd^3+^	sTPATCN	∼33,000^d^		∼2	7	∼0.36	∼18.3	>50	(45)
Er^3+^	CaWO_4_	∼19	∼125	∼5	3.5		∼7 (50)^e^		(11)
Yb^3+^^c^	Y_2_SiO_5_	∼115	∼500	∼4	5	∼7	∼10 (73)^e^	∼9	(18)
Yb^3+^^c^	trensal^b^	∼500	∼600	∼3.8	5	∼0.4	∼0.5	20	(46)

a T* corresponds to the temperature at which *T*_m_ is limited by *T*_1_ given by *T*_m_/*T*_1_ = 1.

b Molecular complex.

c Value reported for ^171^Yb.

d For Gd^3+^, the excited state corresponds to the SOC multiplet and not a CF excited state. Given the ^8^S_7/2_ GS, the CF splitting is usually very small. This minimizes mixing from the excited ^6^P_7/2_ and ^6^D_7/2_ SOC multiplets and has been proposed to suppress decoherence due to the vanishing orbital angular momentum.

e The values in the parenthesis correspond to the maximum value reported but measured at a lower temperature of 2–3 K. For a more direct comparison, the table is constructed with values reported at the base temperature of this study at *T* = 5 K.

While the two host lattices BZO and BSO are isostructural, they exhibit very different *T*_1_ relaxation rates—particularly in the *T* < 12 K regime, indicating that an additional decoherence mechanism besides the SD from neighboring spin-flips is active. In order to understand the decoherence mechanism, a three-pulse stimulated echo technique (π/2 – τ – π/2 – *T*_*W*_ – π/2 – τ – *echo*) and *T* = 5 K is used. Fitting the echo decay to a combination of SD linewidth (Γ_SD_) and relaxation time, a *T*_1_^SD^ = 0.7 and 1.8 ms are obtained for **2Pr:BSO** and **2Pr:BZO**, respectively. The extracted *T*_1_^SD^ values are half of those obtained from the inversion recovery measurements. This difference indicates that besides the spin-flip process, spin-flip flops from neighboring nuclear spins are active as well. The fast Fourier transform (FFT) of the time domain data of **2Pr:BSO** measured with τ = 120 ns clearly shows a peak ≈2.8 MHz, corresponding to the Larmour frequency of ^135^Ba (*I* = 3/2) in the field measured (Figure 5d). The data is well simulated by coupling between an *S* = 1/2 electron and the ^135^Ba nuclei, yielding a hyperfine coupling of *A*_iso_^135_Ba_^ = 0.8 MHz, as shown in Figure 5d.

**Figure 6:**
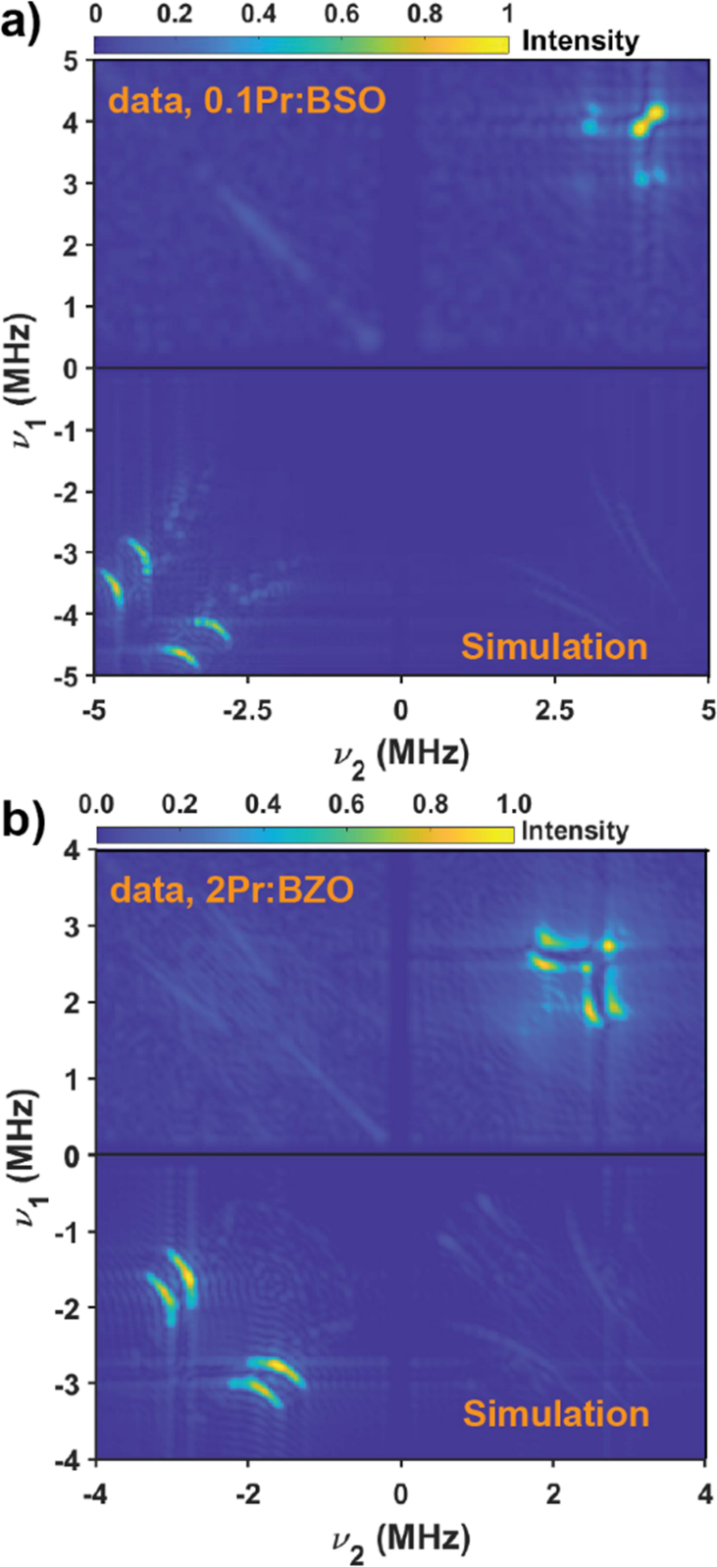
(a) HYSCORE data (top) and simulation (bottom) for **0.1Pr:BSO** measured at μ_0H_ = 901 mT (b) HYSCORE data (top) and simulation (bottom) for **2Pr:BZO** measured at μ_0H_ = 530.8 mT. All data was measured at *T* = 5 K.

Additionally, Hyperfine Sublevel Correlations (HYSCORE) spectroscopy further resolves the coupling to the surrounding Ba nuclei. Both host lattices show two sets of two sharp peaks, which can be simulated by coupling to both ^135^Ba and ^137^Ba (*I* = 3/2) nuclei, yielding *A*_∥_^Ba^ = 0.8 MHz and *A*_⊥_^Ba^ = 1.8 MHz for both nuclei and quadrupolar contributions of *Q*^Ba^ = 3.5 for both nuclei as shown in Figure 6a,b. The key difference between the host lattices is the presence of nuclear spin bearing ^117,119^Sn nuclei (*I* = 1/2) in BSO and ^91^Zr nuclei (*I* = 5/2) in BZO in the surrounding bath of ^141^Pr^4+^. It is possible that the large nuclear spin of ^91^Zr explains the faster *T*_1_ relaxation rate for BZO compared to that for BSO, leading to a faster decoherence.

One of the key properties of ^141^Pr^4+^ is the unusually large hyperfine interaction of ∼2000 MHz. Among the RE elements, holmium metal exhibits the largest hyperfine interaction with *A*_*J*_^Ho^ = 6500 MHz, followed by praseodymium metal with *A*_*J*_^Pr^ = 4500 MHz.^52^ Such large hyperfine coupling interactions have been attributed to the polarization of conduction electrons by s–d mixing minimizing SOC.^52^ A similar argument has been invoked for a very large hyperfine interaction of *A*_iso_^Lu^2+^^ ≈ 3500 MHz observed in Lu^2+^ molecular complexes where the spin bearing d orbitals undergoes symmetry-allowed mixing with s orbitals minimizing SOC.^22^ However, in the solid-state, RE ions, when doped in wide band-gap host lattices, exhibit a significantly reduced hyperfine interaction, as evidenced for Ho^3+^:LiYF_4_ with *A*_iso_ ≈ 800 MHz^53^ or in molecules like Ho^3+^ polyoxometalates with *A*_iso_ ≈ 700 MHz.^54,55^ This large reduction of the hyperfine interaction necessitates the need to understand the origin of the very large hyperfine coupling in ^141^Pr^4+^ and a comparison of the hyperfine interaction of 4f^1 141^Pr^4+^ with 4f^2 141^Pr^3+^.

However, since Pr^3+^ is a non-Kramers ion, it is often EPR silent, at least in the X-band. Therefore, specific-heat measurements were employed. The heat-capacity *C*_N_ arising from a discrete set of 2*I* + 1 hyperfine energy levels *W*_*m*_ (*m* = *I*, ..., –*I*) occurs as a Schottky anomaly with a peak or maximum at a temperature *T* ≃ ⟨Δ*W*⟩_av_/*k*, where ⟨Δ*W*⟩_av_ is the mean spacing of the energy levels and *k* is the Boltzmann constant. The Hamiltonian for the hyperfine Schottky contribution can be written as^56^
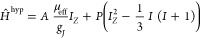
3where *A* is the hyperfine interaction constant, *I*_*z*_ is the expectation values of *I*, μ_eff_ is the saturated magnetic moment, *g*_*J*_ is the landau *g*-factor, and *P* is the quadrupolar contribution. *P* for ^141^Pr is usually three to four orders of magnitude smaller than *A* and therefore can be neglected. Simply, eq 3 can be written as a function of *A* and μ_eff_. By fitting the observed Schottky anomaly in the specific heat data arising from thermal depopulation of the hyperfine spin levels, experimental values of *A* can be extracted.

Heat-capacity measurements on Pr^3+^:LnCl_3_ yield *A*^Pr^3+^ : LnCl_3_^ ≃ 1089 MHz, significantly less than the Pr metal as expected.^57^ Following a similar approach, the heat capacity of **2Pr:BSO** at μ_0_*H* = 7 and 14 T was measured (Figure 5e). Below 1 K, an upturn in specific heat is observed, which is attributed to nuclear Schottky contribution. By fitting the data to eq 3, an *A*^Pr^4+^^ ≃ 1800 MHz and a moment of ∼ 0.6 μ_B_ are extracted, which are consistent with X-band EPR (see Supporting Information for full fitting details). The *A*^Pr^4+^^ value obtained is almost twofold greater than the value reported for Pr^3+^ : LnCl_3_ or other oxide host lattices like Pr^3+^:Y_2_O_3_^15^(*A*^Pr^3+^ : Y_2_O_3_^ ≃ 800 MHz, measured using spectra hall burning). On increasing the oxidation state from Pr^3+^ to Pr^4+^, two antagonistic effects compete to drive the observed hyperfine interaction with the increased nuclear charge, leading to a larger value which is diminished by the enhanced 4f metal–ligand covalency. In this system, the increase in nuclear charge appears to dominate and lead to the significant enhancement in hyperfine interaction.^58^

**Figure 7:**
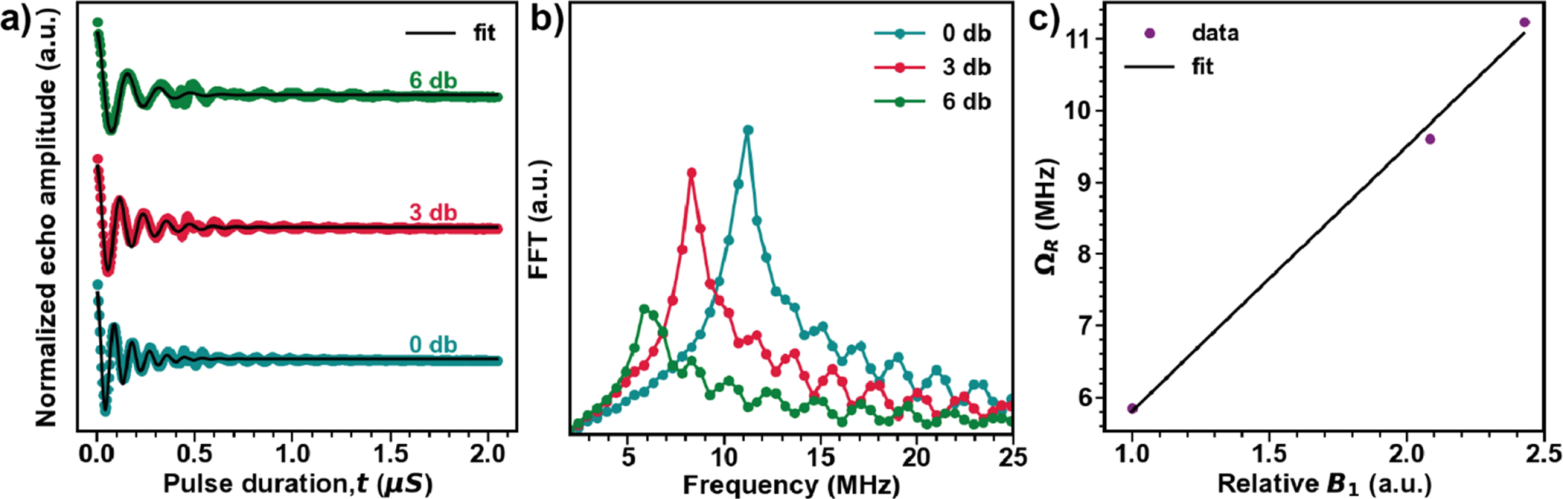
(a) Rabi oscillations for **0.1Pr:BSO** measured at *T* = 5 K at different microwave attenuation. The black lines correspond to fits to the data as described in the text. (b) Frequency domain data for the nutation experiment. (c) *B*_1_ dependence of the Rabi frequency, Ω_R_. The solid line is a linear fit, emphasizing the relationship *B*_1_, where *P* is the microwave power.

Given the relatively long *T*_m_, coherent spin manipulations can be performed, as demonstrated by the observation of Rabi oscillations for **0.1Pr:BSO** in transient nutation experiments (Figure 7a). The damping oscillations were fit with the “on-resonance” transient nutation following 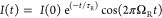
4where τ_R_ is the damping time and Ω_R_ is the Rabi frequency. The corresponding fits are shown in Figure 7a, yielding τ_R_ ≈ 0.2 μs at 0 dB microwave power (which is significantly less than the phase memory time *T*_m_^**0.1 Pr:BSO**^ due to homogeneous and inhomogeneous broadening mechanisms). The FFT of the time domain data yields Ω_R_, consistent with the values obtained by fitting to eq 4 (Figure 7b). The linear relationship between Ω_R_ and relative amplitude *B*_1_ as shown in Figure 7c establishes with certainty the provenance of the observed nutations as Rabi oscillations opposed to coherence transfer from the central spin to the dense bath of nuclear spins in the surroundings. The number of Rabi oscillations given by *N*_c_ = τ_R_(*c*)Ω_R_ with *N*_c_^**0.1 Pr:BSO**^ ≈ 15 compares well with the value reported for ^167^Er^3+^ doped in CaWO_4_. The qubit figure of merit given by *Q*_M_ = 2Ω_R_*T*_m_ reaches up to ∼400 for **0.1Pr:BSO** at *T* = 5 K, the same order of magnitude as other RE qubits except for Er^3+^ which is in the order of 10^4^.

## Conclusions

In conclusion, we show that Pr^4+^ offers the ability to chemically tune the spin–orbit coupled single-ion states as the paradigm shifts from ζ_SOC_ ≫ *V*_CF_ limit to ζ_SOC_ ≪ *V*_CF_ limit. CW X-band EPR measurements of **Pr:BSO** and **Pr:BZO** and CF analysis of the parent material establish the unique single-ion electronic structure of Pr^4+^ with a very small *g*_av_ ≈ 0.6 and a large hyperfine interaction of *A*_iso_ ≈ 1800 MHz. Building on these results, pulsed X-band measurements show the possibility for coherent manipulation of the Pr^4+^ ion with coherence times, reaching a maximum of *T*_1_ = 33 ms and *T*_m_ = 18 μs, with spin dynamics detectable up to T* = 60 K. Therefore, in this tetravalent RE qubit, we have demonstrated long phase memory times exceeding most trivalent RE qubit systems via an alternative approach by employing the large CF energy scale of Pr^4+^ with a vanishing orbital angular momentum via control of the metal oxidation state. Additionally, our work establishes the IC regime as a potential avenue for designing new RE, actinide, heavy (4d and 5d) transition-metal, and main-group^59,60^-based quantum materials, both in solid-state and molecular systems.

## Supporting Information Available

Complete experimental details and further analysis of the data (PDF)
CW data for BSO (TXT)
CW data for BZO (TXT)
EDFS data for BSO (TXT)
EDFS data for BZO (TXT)
MvH data for BaPrO_3_ (TXT)
MvH calculated for BaPrO_3_ (TXT)
MvT fit for BaPrO_3_ (TXT)
MvT ZFC data for BaPrO_3_ (TXT)
MvT FC data for BaPrO_3_ (TXT)
Nutation 0 db (TXT)
Nutation 3 db (TXT)
Nutation 6 db (TXT)
Nutation FFT 0 db (TXT)
Nutation FFT 3 db (TXT)
Nutation FFT 6 db (TXT)
