# Correction to “Increased Crystal Field Drives Intermediate Coupling and Minimizes Decoherence in Tetravalent Praseodymium Qubits”

**DOI:** 10.1021/jacs.6c12385

**Published:** 2026-07-13

**Authors:** Arun Ramanathan, Eric D. Walter, Martin Mourigal, Henry S. La Pierre

The authors wish to add four references that were inadvertently omitted from the original manuscript. These references provide important background on the electron paramagnetic resonance behavior of Pr^4+^ in related oxide and perovskite systems and should be included in the discussion of the structural and electronic context.

The following references should be added to the reference list:

(1)

Tezuka, K. ; Hinatsu, Y. Electron Paramagnetic Resonance Spectra of Pr^4+^ Ions Doped in Pyrochlore-Type Compounds La_2_Sn_2_O_7_ and La_2_Zr_2_O_7_
. J. Solid State Chem. 1999, 143, 140–143.10.1006/jssc.1998.8106

(2)

Hinatsu, Y. ; Wakeshima, M. ; Edelstein, N. ; Craig, I. Electron Paramagnetic Resonance Spectrum of Pr^4+^ in Sr_2_CeO_4_
. J. Solid State Chem. 1999, 144, 20–24.10.1006/jssc.1998.8047

(3)

Hinatsu, Y. Magnetic Susceptibilities and Electron Paramagnetic Resonance Spectra of Lithium-Rich Lanthanide Oxides Li_8_PrO_6_ and Li_8_TbO_6_
. J. Solid State Chem. 1997, 128, 228–232.10.1006/jssc.1996.7193

(4)

Hinatsu, Y. ; Edelstein, N. Electron Paramagnetic Resonance Spectra of Pr^4+^ Ions Doped in BaMO_3_ (M = Ce, Zr, Sn) and SrCeO_3_
. J. Alloys Compd. 1997, 250, 400–404.10.1016/S0925-8388­(96)­02557-1

These additions do not affect the results, discussion, or conclusions of the original article.”

We would like to add these references to page 4, second column following the statement:

“Figure 4a shows the CW-EPR spectra for **2Pr:BSO** and **2Pr:BZO** measured at *T* = 5 K, revealing a six-line pattern due to the unpaired electron and its hyperfine coupling with the *I* = 5/2 ^141^Pr isotope.”

