# Supplementary material for: Increased Crystal Field Drives Intermediate Coupling and Minimizes Decoherence in Tetravalent Praseodymium Qubits
Source: J Am Chem Soc. 2023 Aug 1;145(32):17603–12. doi: 10.1021/jacs.3c02820 (PMC10436280; doi:10.1021/jacs.3c02820)
Supplement: Supplementary file 1 — ja3c02820_si_001.pdf [file ja3c02820_si_001.pdf]

Increased Crystal Field Drives Intermediate Coupling and Minimizes Decoherence in Tetravalent Praseodymium Qubits

Arun Ramanathan, [a] **Eric D. Walter,** [b] **Martin Mourigal,** [c] **and Henry S. La Pierre** *[a,d]

[a] School of Chemistry and Biochemistry, Georgia Institute of Technology, Atlanta, GA, 30332, USA.

[b] Environmental Molecular Sciences Laboratory, Pacific Northwest National Laboratory, Richland, Washington 99352, United States.

[c] School of Physics, Georgia Institute of Technology, Atlanta, GA 30332, USA.

[d] Nuclear and Radiological Engineering and Medical Physics Program, School of Mechanical Engineering, Georgia Institute of Technology, Atlanta, Georgia 30332, USA.

1. Materials Synthesis. **2**
2. Powder X-ray diffraction (PXRD). **3**
3. Physical property measurements. **3**
4. Crystal field analysis of Pr^{4+} in $BaPrO_3$ **4**
5. Specific heat analysis of the Schottky anomaly. **5**
6. Electron paramagnetic resonance. **6**
7. References. **12**

1. Materials Synthesis.

All reagents were handled in a N_2 filled glove box (Vigor) with O_2 < 0.1 ppm and H_2O < 0.1 ppm. $BaCO_3$ (99.5%, Alfa Aesar), Pr_6O_{11}(> 99.5%, Alfa Aesar), SnO_2 (> 99.9%, Alfa Aesar), and ZrO_2 (> 99.9%, Alfa Aesar) were used as starting materials. The metal oxides and $BaCO_3$ powders were dried by heating to 500°C for ~12 h with a heating rate of 10°C/min in a box furnace (using alumina crucibles) under ambient atmosphere. The reagents were then cooled with the furnace off to ~120°C, and then cooled to room temperature in the antechamber of the glovebox under vacuum. These dried reagents were stored in amber bottles in the glove box. An MTI-KSL-1100X-S-UI-LD furnace was used. All crucibles were purchased from MTI.

Polycrystalline powder samples of $BaPrO_3$ were synthesized using traditional solid-state methods by intimately mixing $BaCO_3$ and Pr_6O_{11} in molar ratio 1:1 (Ba:Pr), using an agate mortar inside the glove box. The powder mixtures were pressed in to 15 mm diameter pellets outside the glovebox. The samples were fired under a flow of O_2 in tube furnace (quartz tubes with a diameter of 55 mm was used). The O_2 flow was controlled using a regulator set to 2 psig and an oil bubbler at the end of the line to ~1 bubble every 2-3 sec. The pellets were placed on alumina boats and placed at the center of the quartz tube (lining up with the center of the heating zone in the furnace). The line was then purged with O_2 for ~5 min. The firing was performed at 1100°C for 24 h with a cooling/heating rate of 3°C/min. O_2 flow was stopped 30 min after the furnace cooled to room temperature. The samples were taken out of the quartz tubes in air and placed into the antechamber of the glovebox as quickly as possible in order minimize contact with ambient atmosphere. The samples were ground, and the above procedure was repeated twice to yield phase pure material (see section 2).

Pr doped in $BaMO_3$ (M = Zr, Sn) were synthesized in a manner similar to that described for $BaPrO_3$ by intimately mixing $BaCO_3$, SnO_2 (ZrO_2), and Pr_6O_{11} in molar ratio 1:0.98:0.02 (Ba:M:Pr) for 2% loading. A similar procedure was followed for 0.1% loading. Phase purity was established by PXRD (see section 2).

2. Powder X-ray diffraction (PXRD).

Laboratory powder X-ray diffraction (PXRD) was collected on a PANalytical X'Pert PRO Alpha-1 diffractometer with Cu Kα source in reflection geometry equipped with a fixed divergence slit of 1/8", a convergence slit of 1/4" and a working radius of 240 mm. The samples were homogenized by finely grinding them inside the glove box using an agate mortar for about ~15 min. To avoid the exposure of sample to atmospheric air, PANalytical domed sample holder equipped with stainless steel base and a polycarbonate dome with a 70% X-ray transmission. A 2θ range of 5 - 85° was used with a scan speed of 5 s and a step size of 0.1.

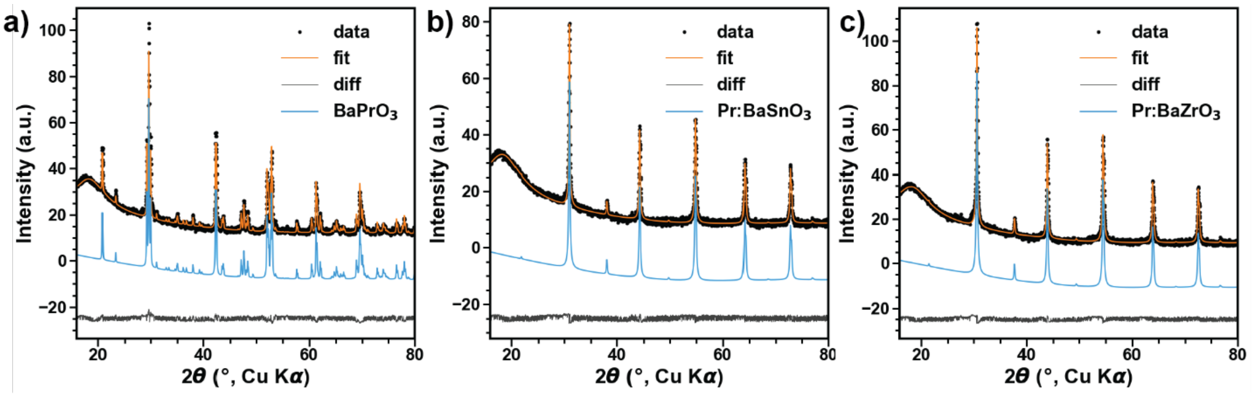

Figure S1. (a) **$BaPrO_3$** (b) **2Pr:$BaSnO_3$** (c) **2Pr:$BaZrO_3$**. Data is shown in black dots, Rietveld refinements[1] in orange, the corresponding phases in blue, and the difference curves in grey. The broad hump near 2θ = 20° corresponds to polycarbonate dome background from the sample holder. All data was collected at *T*=300 K. Quantitative Rietveld refinements to the laboratory XRD data were carried out using Bruker TOPAS 5 suite.[2]

Table S1. Crystal structure information.

	$BaPrO_3$	Pr:$BaSnO_3$	Pr:$BaZrO_3$
Space group	*Pnma*	$Pm\bar{3}m$	$Pm\bar{3}m$
Point group	D_{2d}	O_h	O_h
a (Å)	6.206(8)	4.115(8)	4.197(2)
b (Å)	8.72(5)	4.115(8)	4.197(2)
c (Å)	6.179(8)	4.115(8)	4.197(2)
α (°)	90	90	90
β (°)	90	90	90
γ (°)	90	90	90
$d_{Pr\text{-}Ba}$ (Å)	3.407(3)	3.487(6)	
$d_{Pr\text{-}M}$ (Å)	3.407(3)	3.487(6)	

3. Physical property measurements.

The d.c. magnetic susceptibility measurements and isothermal magnetization measurements were performed using a Quantum Design MPMS3. The sample was sealed in a plastic capsule on a low-background brass holder. The heat capacity was measured at different magnetic fields using a Quantum Design Physical Properties Measurement System. For 0.09 K < T < 4 K a Quantum Design dilution fridge insert was employed. The sample was ground together with 32% by mass of silver and pressed into pellets. A small pellet yielding 7.62 mg was used for the measurement.

4. Crystal field analysis of Pr^{4+} in $BaPrO_3$

We begin by constraining the CF Hamiltonian for Pr^{4+} in $BaPrO_3$ under a perfect O_h CF while the real symmetry is C_{2h} in order to reduce the number of parameters for fitting, $\hat{H}_{CF} = B_4^0\hat{O}_4^0 + B_4^4\hat{O}_4^4 + B_6^0\hat{O}_6^0 + B_6^4\hat{O}_6^4$, where B_4^4, and B_6^4 were constrained under O_h symmetry. Also, B_4^0 was constrained to be > 0 as expected for a six-coordinate system. The energy scale of the uncommonly large Δ_{CF} in $BaPrO_3$ is comparable in magnitude to ζ_{SOC} of Pr^{4+} and requires an intermediate coupling scheme where we diagonalize the $\hat{H}_{CF}$ with the entire set of 14*LS* basis states as described elsewhere.[3] The Hamiltonian was diagonalized using PyCrystalField.[4] Initial guesses for the steven's coefficients B_4^0 and B_6^0 were obtained in the $|j, m_j\rangle$ basis by setting the first excited state to E^2 = 250 meV. We note here that, point change-based estimation of Steven's coefficients is not appropriate for Pr^{4+} given the anomalously large Pr-4*f*/O-2*p* covalency.[3] With the initial guesses for B_4^0 and B_6^0, we start fitting the susceptibility data (T>50 K, to avoid the region with short-range correlations) and eigen energies and degeneracies to the Hamiltonian $\hat{H}_{CF}$. With the newly estimated values for B_4^0 and B_6^0, we begin to relieve the cubic constraints on B_4^4 and B_6^4, to account for the slight distortion from perfect O_h symmetry. Again, fitting to the susceptibility and eigen energies and degeneracies yields newly estimated values for the stevens coefficients. Although, the true symmetry of $BaPrO_3$ requires |m| = 2,6 in addition to |m| = 0,4 (in B_n^m coefficients), any mixing induced by these parameters would not induce any further loss of degeneracy and hence their effects can be parameterized with |m| = 0,4 parameters. Therefore, we use the truncated Hamiltonian $\hat{H}_{CF}$ described above. The final fitting was carried out by providing different weights to susceptibility and eigen energies. The final fit parameters and results are provided in Table S2. This yields a set of KD's with the ground state wavefunction given as $|\Gamma_7^{\pm}\rangle = -0.262\,|\mp 3, \pm\frac{1}{2}\rangle + 0.365\,|\mp 2, \mp\frac{1}{2}\rangle + 0.35\,|\pm 1, \pm\frac{1}{2}\rangle - 0.822\,|\pm 2, \mp\frac{1}{2}\rangle$. This yields a slight easy-axis anisotropic g with g_{xy} = 0.65 and g_z = 0.76. Even after relieving the cubic constraints, the value obtained for B_4^4 = 2.26 is close to 5*B_4^0 = 2.4 and B_6^4 = 0.063 is close to -21*B_6^0 = 0.077. The closeness of these values further show that the PrO_6 exhibits only a slight distortion from a perfect cubic symmetry.

Table S2. Fit parameters for Pr^{4+} in $BaPrO_3$.

	Γ_7 (in SOC limit)	$BaPrO_3$	
B_4^0 (meV)	*	0.48	
B_4^4 (meV)	*	2.26	
B_6^0 (meV)	*	-0.0037	
B_6^4 (meV)	*	0.063	
ζ_{SOC} (meV)	112	113.9	
g_{av}	~1.4	~0.6	
A[a]	0.352	0.261	
B[a]	0.215	0.365	
C[a]	0.454	0.348	
D[a]	0.79	-0.823	
KD1 (meV)	*	0	0
KD2 (meV)	*	249.5[b]	256[c]
KD3 (meV)	*	252[b]	262[c]
KD4 (meV)	*	389[b]	433[c]
KD5 (meV)	*	655[b]	662[c]
KD6 (meV)	*	657[b]	673[c]
KD7 (meV)	*	818[b]	792[c]

[a] coefficient for the ground state wave function as described in the main text.
[b] Experimentally observed Eigen energies.
[c] Calculated from the CF model.

Table S3. Eigenvectors and Eigenvalues for Pr^{4+} in $BaPrO_3$.

***E* (meV)**	**0**	**256.29**	**262.4**	**433.6**	**662.4**	**673**	**792.2**
$\|-3,-\frac{1}{2}\rangle$	0	0	0	0	0	0	0.63
$\|-3,+\frac{1}{2}\rangle$	-0.261	0	0	0	0.447	0	0
$\|-2,-\frac{1}{2}\rangle$	0.365	0	0	0	0.322	0	0
$\|-2,+\frac{1}{2}\rangle$	0	0.462	0	-0.053	0	0	0
$\|-1,-\frac{1}{2}\rangle$	0	0.016	0	-0.537	0	0	0
$\|-1,+\frac{1}{2}\rangle$	0	0	0.855	0	0	0.15	0
$\|0,-\frac{1}{2}\rangle$	0	0	-0.461	0	0	0.656	0
$\|0,+\frac{1}{2}\rangle$	0	0	0	0	0	0	0.597
$\|+1,-\frac{1}{2}\rangle$	0	0	0	0	0	0	0.496
$\|+1,+\frac{1}{2}\rangle$	0.348	0	0	0	0.768	0	0
$\|+2,-\frac{1}{2}\rangle$	-0.823	0	0	0	0.326	0	0
$\|+2,+\frac{1}{2}\rangle$	0	0.505	0	0.712	0	0	0
$\|+3,-\frac{1}{2}\rangle$	0	-0.729	0	0.448	0	0	0
$\|+3,+\frac{1}{2}\rangle$	0	0	-0.236	0	0	-0.74	0

5. Specific heat analysis of the Schottky anomaly.

The total heat capacity is given as $C_p = C_L + C_M + C_N + C_{CF}$, where C_L, C_M, and C_N are the lattice, magnetic, nuclear specific heats respectively, and C_{CF} is CF contribution to the specific heat from the first excited state. In the temperature range measured, lattice contributions are negligible with $C_L \approx 0$. Furthermore, at 2% dilution, we expect negligible magnetic interactions between the Pr ion making $C_M \approx 0$. Furthermore, given the first CF excitation is ≈ 250 meV, CF contribution to specific-heat can be neglected. Therefore, the specific-heat less than ~3 K is dominated only by nuclear Schottky contributions. Below 1.0 K, there is an upturn which can be associated with nuclear spin degrees of freedom. The Schottky anomaly was modeled using equation 4 in the main text. The Hamiltonian was diagonalized using PyNuclearSchottky.[5]

6. Electron paramagnetic resonance.

Electron paramagnetic resonance (EPR) experiments were carried out on a Bruker E580 X-band spectrometer. Samples (~90-100 mg) were contained in 4 mm OD quartz tubes that were filled in an inert atmosphere glovebox and flame sealed with an oxy-hydrogen torch. For pulsed EPR experiments the spectrometer was outfitted with an MD-5 dielectric resonator and an Oxford liquid helium continuous flow cryostat which maintained the temperature at 5-20 K.

The T_1 of $^{141}Pr^{4+}$ ions in the temperature range 5-60 K was studied using the inversion-recovery method ($\pi - \tau_r - \frac{\pi}{2} - \tau_e - \pi - \tau_e - echo$), where τ_r is swept ($\tau_e = 200$ ns). These experiments, at an applied field of B_0= 592.7 mT (**2Pr:BSO**) and 530.8 mT (**2Pr:BZO**), focus on the field of the largest intensity echo. The resulting saturation recovery traces were fit with a standard stretched mono-exponential function.

In order to understand the decoherence mechanism, a three-pulse stimulated echo technique ($\frac{\pi}{2} - \tau - \frac{\pi}{2} - T_W - \frac{\pi}{2} - \tau - echo$) and T = 5 K is used.

T_m were measured through two-pulse Hahn echo measurements, τ = 120 ns

Typical parameters for HYSCORE experiments with a $\frac{\pi}{2} - \tau - \frac{\pi}{2} - t_1 - \pi - t_2 - \frac{\pi}{2}$ sequence were: $\frac{\pi}{2}$ = 24 ns, π = 48 ns, τ = 200 ns with 350 delays of t_1/t_2 in 12 ns steps. The recycle delay was ~ 3500 μs and typically 30 shots were collected for each delay. T_1 was extracted from the measurements by fitting a standard stretched monoexponential [I(t) = I_0+ A·exp(t/T_1)^b] to the data. T_m was extracted from the measurements by fitting a standard monoexponential [I(t) = I_0+ A·exp(t/T_m)] to the data.

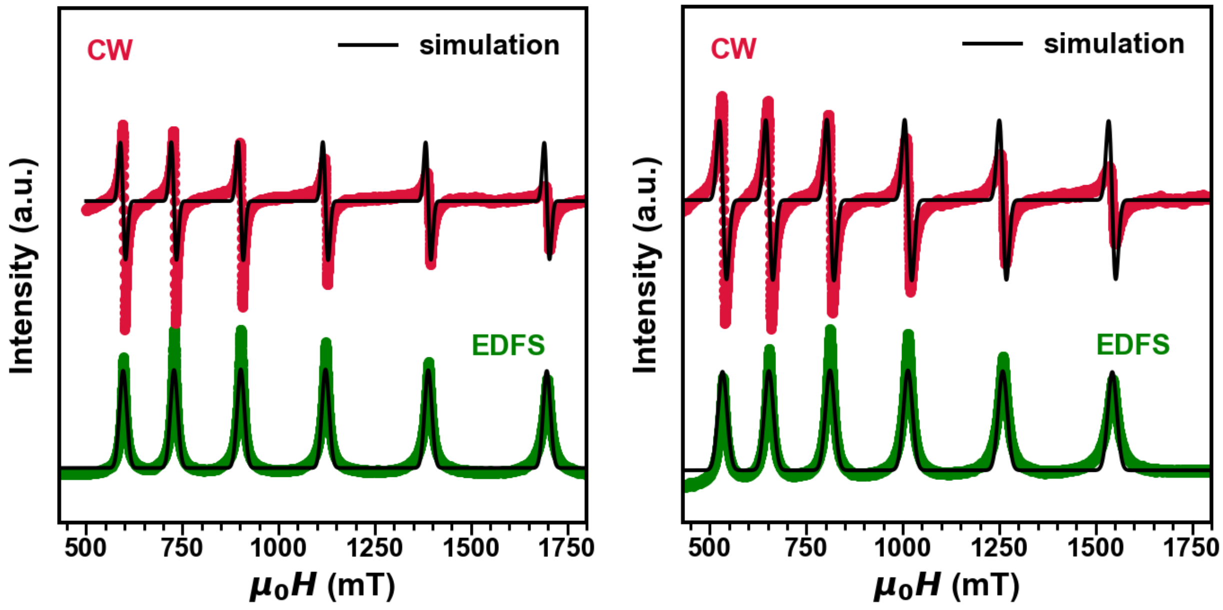

Figure S2. (a) CW and EDFS spectra plotted together for **2Pr:BSO**. (b) CW and EDFS spectra plotted together for **2Pr:BZO**

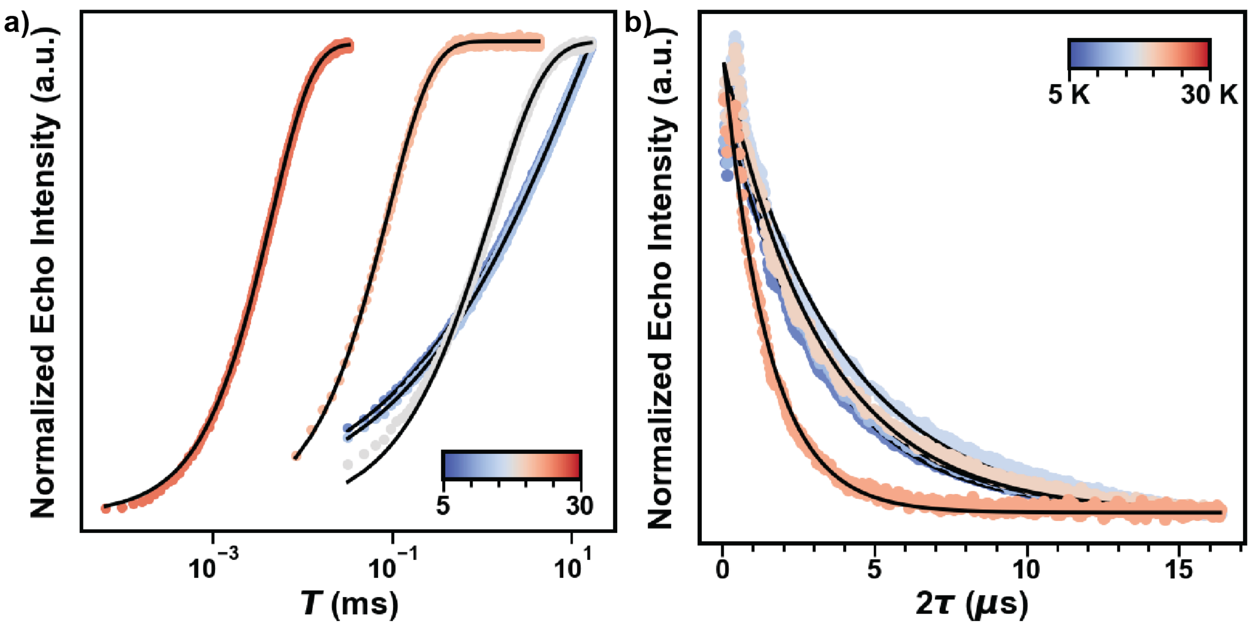

Figure S3. (a) Inversion recovery traces and the corresponding fits used to extract T_1 for **2Pr:BSO** measured at different temperatures. (b) Echo decays and the corresponding fits used to extract T_m for **2Pr:BSO** measured at different temperatures.

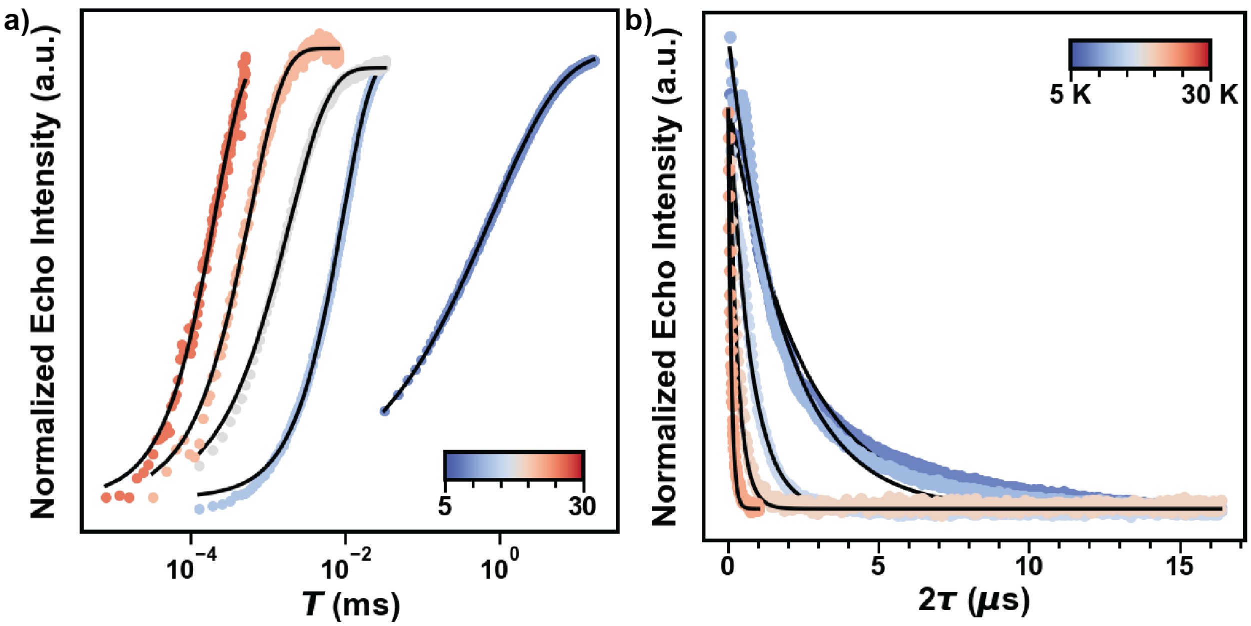

Figure S4. (a) Inversion recovery traces and the corresponding fits used to extract T_1 for **2Pr:BZO** measured at different temperatures. (b) Echo decays and the corresponding fits used to extract T_m for **2Pr:BZO** measured at different temperatures.

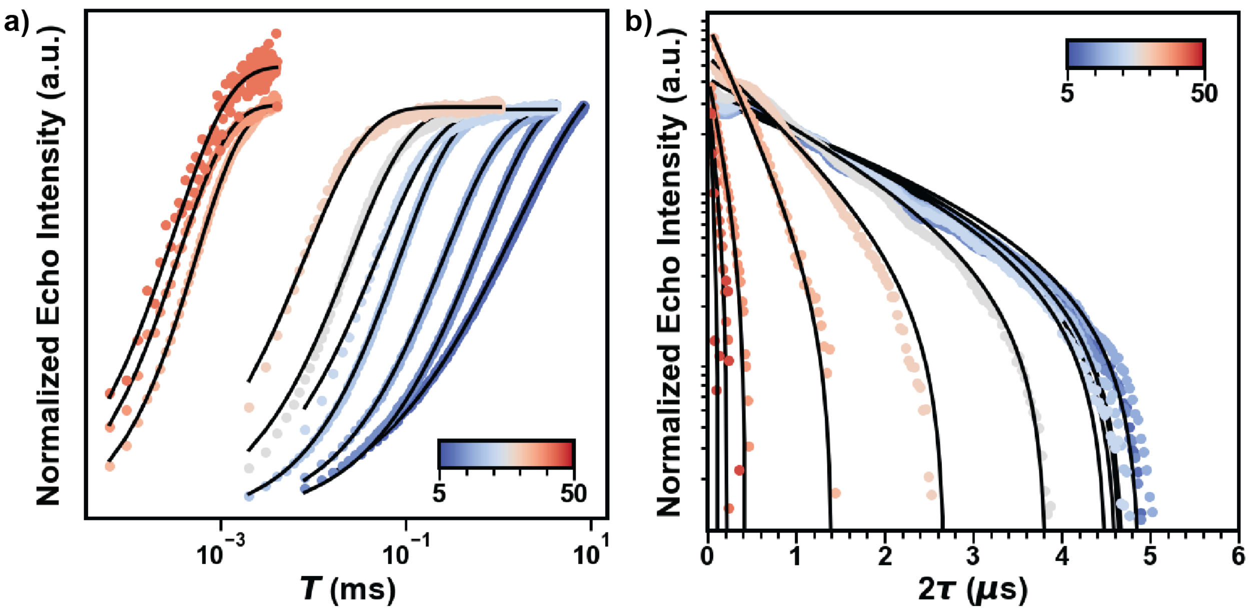

Figure S5. (a) Inversion recovery traces and the corresponding fits used to extract T_1 for **0.1Pr:BZO** measured at different temperatures. (b) Echo decays and the corresponding fits used to extract T_m for **0.1Pr:BZO** measured at different temperatures.

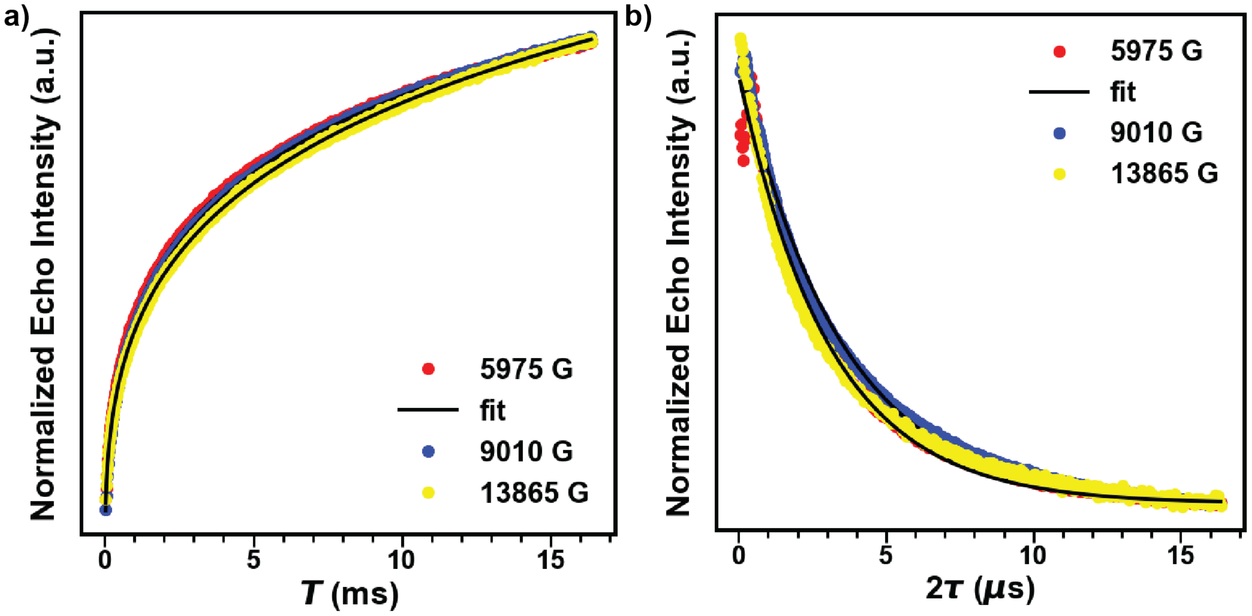

Figure S6. (a) Inversion recovery traces and the corresponding fits used to extract T_1 for **2Pr:BSO** measured at different fields. (b) Echo decays and the corresponding fits used to extract T_m for **2Pr:BSO** measured at different fields.

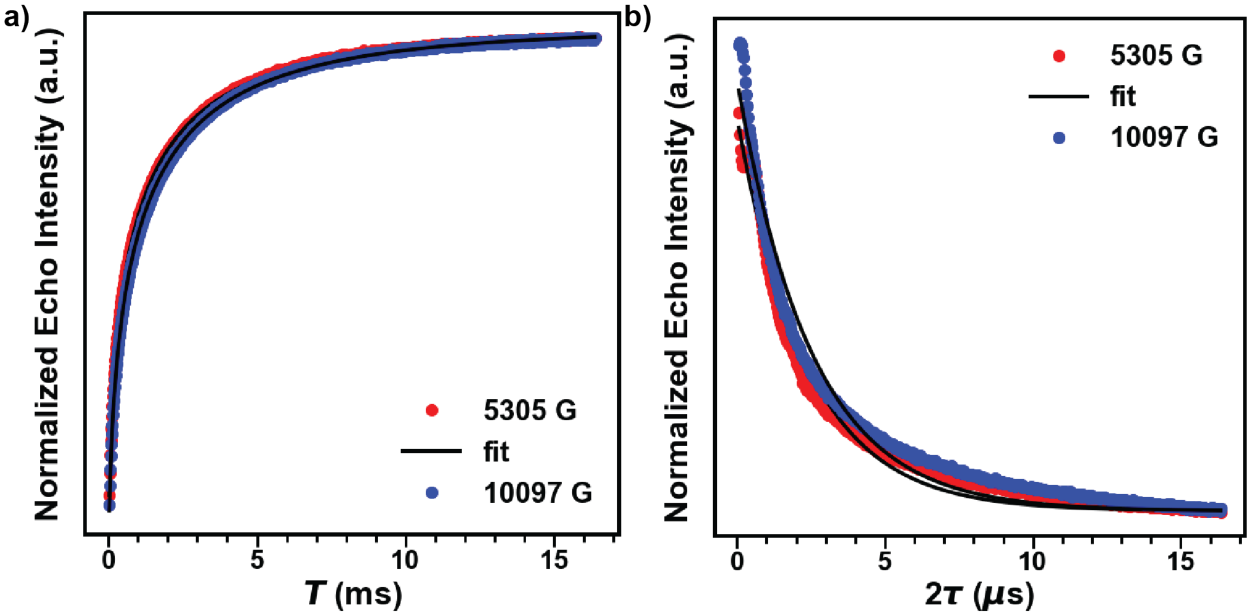

Figure S7. (a) Inversion recovery traces and the corresponding fits used to extract T_1 for **2Pr:BZO** measured at different fields. (b) Echo decays and the corresponding fits used to extract T_m for **2Pr:BZO** measured at different fields.

Table S4. Fit parameters extracted from the pulsed X-band EPR measurements. .

T (K)	**0.1Pr:BSO**		**0.1Pr:BZO**		**2Pr:BSO**		**2Pr:BZO**	
	T_1 (ms)	T_m (μs)	T_1 (ms)	T_m (μs)	T_1 (ms)	T_m (μs)	T_1 (ms)	T_m (μs)
5	33	18	15	2.5	13	3.	0.7	2.3
6	35	-	6	2.4	-	-	-	-
7	36	18	2	2.7	-	-	-	-
8	37	-	0.8	-	-	-	-	-
9	-	-	0.4	2.4	-	-	-	-
10	35	18	0.2	2.3	13	3.2	0.09	1.8
12	37	-	0..08	1.7	-	-	-	-
15	1.5	4.3	-	0.9	1.2	3.1	0.01	0.7
16	0.8	-	-	-	-	-	-	-
17	0.4	3.6	-	-	-	-	-	-
18	0.2	-	-	-	-	-	-	-
19	0.1	-	-	-	-	-	-	-
20	0.09	3.2	0.005	0.3	0.08	3.1	0.005	0.2
22	0.04	-	-	-	-	-	-	-
25	0.01	2.3	0.0034	0.17	-	-	-	-
30	0.004	1.4	0.0031	0.1	0.004	1.4	0.002	0.099
35	0.001	0.9	-	-	-	-		
40	0.0008	0.6	0.0002	0.004	-	-		
45	0.0006	0.4	-	-	-	-		
50	0.0004	0.2	-	-	-	-		
60	0.0001	0.09	-	-	-	-		

Table S5. Fit parameters extracted from the pulsed X-band EPR measurements for different hyperfine transitions.

**2Pr:BSO**			**2Pr:BZO**		
Field (mT)	T_1 (ms)	T_m (μs)	Field (mT)	T_1 (ms)	T_m (μs)
597.5	33	18	530.5	15	2.5
901.0	32	19	1009.7	14	2.2
1386.5	34	21			

Table S6. Fit parameters extracted from the X-band EPR measurements.

**2Pr:BSO**						**2Pr:BZO**					
g_{av}		A_j (MHz)		lwpp		g_{av}		A_j (MHz)		lwpp	
CW	EDFS	CW	EDFS	CW	EDFS	CW	EDFS	CW	EDFS	CW	EDFS
0.5791	0.5787	1771.12	1768.94	13.69	18.858	0.6388	0.6385	1788.92	1790.85	18.469	23.647

The three pulse stimulated echo data decays according to a function of both T_1 and spectral diffusion linewidth (Γ_{SD}) as:

$$\frac{A\ (\tau, T_W)}{A_0} = exp\ [-(\frac{T_1}{T_W} + 2\pi\tau\Gamma_{eff})$$

Where, Γ_{eff} is a combination of SD and ID.

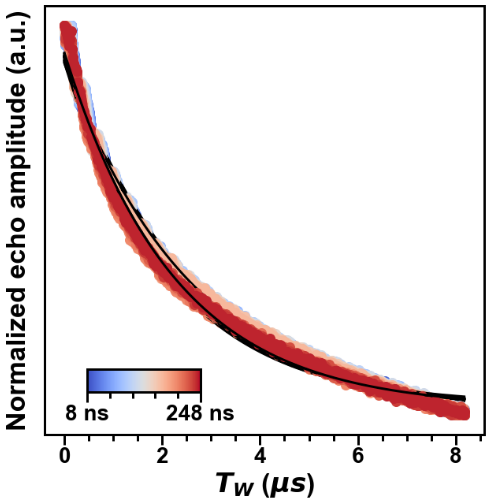

Figure S8. (a) 3 pulse echo decays for **2Pr:BSO**.as a function of T_W

7. Spin lattice relaxation.

The SLR for T > 10 K in 0.1Pr:BSO was fit to a combination of Raman and local based on:

$$\frac{1}{T_1} = a_{loc} * (exp(\theta_E/T)./(exp(\theta_E/T) - 1)\hat{}2);$$

For the local mode and

$$\frac{1}{T_1} = a_{ram} * (\frac{T}{\theta_D})J_8(\frac{\theta_D}{T});$$

Where J_8 is the transport integral.

8. References.

1. Rietveld, H., A profile refinement method for nuclear and magnetic structures. *J. Appl. Crystallogr.* **1969,** *2* (2), 65-71.
2. Coelho, A. A., TOPAS and TOPAS-Academic: an optimization program integrating computer algebra and crystallographic objects written in C++. *J. Appl. Crystallogr.* **2018,** *51* (1), 210-218.
3. Ramanathan, A.; Kaplan, J.; Sergentu, D.-C.; Branson, J. A.; Ozerov, M.; Kolesnikov, A. I.; Minasian, S. G.; Autschbach, J.; Freeland, J. W.; Jiang, Z., Chemical Design of Electronic and Magnetic Energy Scales in Tetravalent Praseodymium. *arXiv preprint arXiv:2212.10401* **2022**.
4. Scheie, A., PyCrystalField: software for calculation, analysis and fitting of crystal electric field Hamiltonians. *J. Appl. Crystallogr.* **2021,** *54* (1), 356-362.
5. Scheie, A. https://github.com/asche1/PyNuclearSchottky.
